# 非血缘脐血干细胞移植和同胞外周血干细胞移植免疫重建与慢性移植物抗宿主病的研究

**DOI:** 10.3760/cma.j.issn.0253-2727.2021.06.005

**Published:** 2021-06

**Authors:** 娇 王, 田中 潘, 盼盼 黄, 自敏 孙, 怀平 祝

**Affiliations:** 1 中国科学技术大学附属第一医院（安徽省立医院），合肥 230001 The First Affiliated Hospital of University of Science and Technology of China （Anhui Provincial Hospital）, Hefei 230001, China; 2 中国科学技术大学生命科学与医学部血液与细胞治疗研究所，合肥 230036 Institute of Blood and Cell Therapy, Division of Life Sciences and Medicine, University of Science and Technology of China, Hefei 230036, China

**Keywords:** 造血干细胞移植, 脐血移植, 外周血干细胞移植, 免疫重建, 慢性移植物抗宿主病, Hematopoietic stem cell transplantation, Cord blood transplantation, Peripheral blood stem cell transplantation, Immune reconstruction, Chronic graft-versus-host disease

## Abstract

**目的:**

探究恶性血液病患者非血缘脐血干细胞移植（UCBT）和同胞外周血干细胞移植（PBSCT）后免疫重建与慢性移植物抗宿主病（cGVHD）的关系。

**方法:**

以2018年3月至2019年8月在中国科学技术大学附属第一医院行UCBT（96例）和PBSCT（28例）的患者为研究对象。采用流式细胞术检测两组患者移植后第1、3、6、9、12个月外周血免疫细胞，并根据是否发生cGVHD进行分组，探究两种移植类型的免疫细胞重建与cGVHD之间的相关性。

**结果:**

①UCBT组移植后1年中重度cGVHD累积发生率显著低于PBSCT组［9.38％（95％*CI* 3.35％～15.02％）对28.57％（95％*CI* 9.72％～43.50％），*P*＝0.008］；UCBT组移植后2年cGVHD、中重度cGVHD累积发生率均低于PBSCT组［15.60％（95％*CI* 9.20％～23.60％）对32.10％（95％*CI* 15.80％～49.70％），*P*＝0.047；10.40％（95％*CI* 5.30％～17.50％）对28.60％（95％*CI* 13.30％～46.00％），*P*＝0.014］。②UCBT组CD4^+^T细胞计数在移植后第6、9、12个月均高于PBSCT组［59.00（36.70～89.65）×10^7^/L对31.40（18.10～44.00）×10^7^/L，*P*<0.001；71.30（49.60～101.45）×10^7^/L对41.60（25.82～56.27）×10^7^/L，*P*<0.001；83.00（50.17～121.55）×10^7^/L对44.85（31.62～62.10）×10^7^/L，*P*<0.001］，CD4 ^+^T细胞比例始终高于PBSCT组（*P*<0.05）。PBSCT组B细胞计数和比例在移植后第1个月高于UCBT组［0.70（0.30～1.70）×10^7^/L对0.10（0～0.30）×10^7^/L，*P*<0.001；0.45％（0.30％～2.20％）对0.20％（0.10％～0.40％），*P*＝0.002］；UCBT组B细胞计数和比例在移植后第9、12个月高于PBSCT组［53.80（28.00～103.20）×10^7^/L对23.35（5.07～35.00）×10^7^/L，*P*<0.001；21.45％（11.80％～30.45％）对9.00％（3.08％～16.73％），*P*<0.001。66.70（36.97～98.72）×10^7^/L对20.85（7.72～39.40）×10^7^/L，*P*<0.001；22.20％（14.93％～29.68）％对8.75％（5.80％～18.93％），*P*<0.001］。UCBT组调节性B细胞（Breg）计数和比例在移植后第6、9和12个月高于PBSCT组［1.23（0.38～3.52）×10^7^/L对0.05（0～0.84）×10^7^/L，*P*<0.001；5.35％（1.90％～12.20％）对1.45％（0％～7.78％），*P*＝0.002。2.25（1.07～6.71）×10^7^/L对0.12（0～0.77）×10^7^/L，*P*<0.001；6.25％（2.00％～12.33％）对0.80％（0％～5.25％），*P*<0.001。3.69（0.83～8.66）×10^7^/L对0.46（0～0.93）×10^7^/L，*P*<0.001；6.15％（1.63％～11.75％）对1.40％（0.18％～5.85％），*P*<0.001］。③UCBT患者中非cGVHD组的B细胞计数在移植后第6、12个月均高于中重度cGVHD组（*P*＝0.038，*P*＝0.043）；非cGVHD组的B细胞比例在移植后第6个月高于中重度cGVHD组（*P*＝0.049）。UCBT患者中非cGVHD组的Breg细胞计数在移植后第6、9、12个月高于中重度cGVHD组（*P*＝0.006，*P*＝0.028，*P*＝0.050）；非cGVHD组的Breg细胞比例在移植后第9个月高于中重度cGVHD组（*P*＝0.038）。④PBSCT患者中非cGVHD组的B和Breg细胞绝对数和比例与中重度cGVHD组差异无统计学意义。

**结论:**

在免疫细胞重建过程中，UCBT组的Breg细胞高于PBSCT组，并且两种移植类型的非cGVHD组的Breg细胞始终较中重度cGVHD组高，表明Breg细胞能够降低cGVHD的发生，揭示了UCBT组cGVHD发生率较低的可能原因。

## Abstract (en)

**Objective:**

To explore the relationship between the reconstitution of immune cells in patients with hematological malignancies and the occurrence of chronic graft-versus-host disease （cGVHD）after treatment with unrelated cord blood transplantation（UCBT）and sibling peripheral blood stem cell transplantation（PBSCT）.

**Methods:**

A total of 124 patients undergoing allogenic hematopoietic stem cell transplantation （allo-HSCT） in the First Affiliated Hospital of University of Science and Technology of China from March 2018 to August 2019, including 96 patients with UCBT and 28 patients with PBSCT. Peripheral blood immune cells of patients with UCBT and PBSCT were detected at 1, 3, 6, 9, and 12 months after transplantation using flow cytometry, and both UCBT and PBSCT patients were divided into cGVHD and non-cGVHD groups based on whether cGVHD occurred to explore the correlation between the immune cells reconstitution of the two types of transplantation and cGVHD.

**Results:**

① The cumulative incidence of the moderate to severe cGVHD in the UCBT group was significantly lower than that in the PBSCT group［9.38％（95％ *CI* 3.35％–15.02％）*vs* 28.57％（95％ *CI* 9.72％–43.50％）, *P*＝0.008］; the 2-year cumulative incidence of cGVHD and moderate to severe cGVHD in the UCBT group was lower than that in the PBSCT group［15.60％（95％ *CI* 9.20％–23.60％）*vs* 32.10％（95％ *CI* 15.80％–49.70％）, *P*＝0.047; 10.40％（95％ *CI* 5.30％–17.50％）*vs* 28.60％（95％ *CI* 13.30％–46.00％）, *P*＝0.014］. ②The absolute counts of CD4^+^T cells in the UCBT group were higher than those in the PBSCT group at 6, 9, and 12 months after transplantation［59.00（36.70–89.65）×10^7^/L *vs* 31.40 （18.10–44.00）×10^7^/L, *P*<0.001; 71.30（49.60–101.45）×10^7^/L *vs* 41.60（25.82–56.27）×10^7^/L, *P*<0.001; 83.00（50.17–121.55）×10^7^/L *vs* 44.85（31.62–62.10）×10^7^/L, *P*<0.001］; the proportions of CD4^+^T cells in the UCBT group were always higher than those in the PBSCT group（*P*<0.05）. The absolute counts and proportions of B cells in the PBSCT group were higher than those in the UCBT group at the first month after transplantation［0.70（0.30–1.70）×10^7^/L *vs* 0.10（0–0.30）×10^7^/L, *P*<0.001; 0.45％（0.30％–2.20％）*vs* 0.20％（0.10％–0.40％）, *P*＝0.002］; the absolute counts and proportions of B cells in the UCBT group were higher than those in the PBSCT group at 9 and 12 months after transplantation ［53.80（28.00–103.20）×10^7^/L *vs* 23.35（5.07–35.00）×10^7^/L, *P*<0.001; 21.45（11.80–30.45）％ *vs* 9.00％（3.08％–16.73％）, *P*<0.001. 66.70（36.97–98.72）×10^7^/L *vs* 20.85（7.72–39.40）×10^7^/L, *P*<0.001; 22.20％ （14.93％–29.68％）*vs* 8.75％（5.80％–18.93％）, *P*<0.001］. The absolute counts and proportions of regulatory B（Breg）cells in the UCBT group were higher than those in the PBSCT group at 6, 9, and 12 months after transplantation ［1.23（0.38–3.52）×10^7^/L *vs* 0.05（0–0.84）×10^7^/L, *P*<0.001; 5.35％ （1.90％–12.20％）*vs* 1.45％（0–7.78％）, *P*＝0.002. 2.25（1.07–6.71）×10^7^/L *vs* 0.12（0–0.77）×10^7^/L, *P*<0.001; 6.25％（2.00％–12.33％）*vs* 0.80％（0–5.25％）, *P*<0.001. 3.69（0.83–8.66）×10^7^/L *vs* 0.46（0–0.93）×10^7^/L, *P*<0.001; 6.15％（1.63％–11.75％）*vs* 1.40％（0.18％–5.85％）, *P*<0.001］.The absolute counts and proportions of CD3 ^+^ T cells, CD8 ^+^ T cells, and Treg cells in the UCBT group were not significantly different from those in the PBSCT group. ③The absolute counts of B cells in the non-cGVHD group of UCBT patients were higher than those in the moderate to severe cGVHD group at 6 and 12 months after transplantation（*P*＝0.038, *P*＝0.043）; the proportions of B cells in the non-cGVHD group were higher than those in the moderate to severe cGVHD group at 6 months after transplantation（*P*＝0.049）. The absolute counts of Breg cells in the non-cGVHD group of patients with UCBT were higher than those in the moderate to severe cGVHD group at 6, 9, and 12 months after transplantation（*P*＝0.006, *P*＝0.028, *P*＝0.050）; the proportions of Breg cells in the non-cGVHD group were higher than those in the moderate to severe cGVHD group at 9 months after transplantation（*P*＝0.038）. ④The absolute counts and proportions of B and Breg cells in the non-cGVHD group of patients with PBSCT were not statistically different than those in the moderate to severe cGVHD group.

**Conclusion:**

In the process of immune cell reconstitution, the Breg cells in the UCBT group were higher than those in the PBSCT group, and the Breg cells in the non-cGVHD group of the two types of transplantation were always higher than those in the moderate to severe cGVHD group, indicating that Breg cells can reduce the occurrence of cGVHD, revealing the possible reason for the lower incidence of cGVHD in the UCBT group.

异基因造血干细胞移植（allo-HSCT）是治疗多种恶性血液病、实体肿瘤、遗传代谢性疾病、骨髓衰竭性疾病等疾病最有效的手段之一[1]。目前可供选择的移植物主要包括骨髓、外周血和脐血。由于脐血来源丰富、采集过程无伤害、病毒污染概率低、免疫原性弱、发生移植物抗宿主病（GVHD）的风险低以及HLA配型要求相对比较低等优点，脐血造血干细胞移植（UCBT）已被广泛应用于临床[2]–[3]。allo-HSCT后快速免疫重建可以降低感染、继发性肿瘤以及原发病复发的风险，而不同的移植物来源、预处理方案以及GVHD的预防方案等均影响allo-HSCT后淋巴细胞的免疫重建[4]–[7]。因此，分析移植后免疫重建不仅可以预测GVHD等移植相关并发症的发生，而且有助于对患者的长期生存进行早期评估，进而为选择最佳治疗方案提供依据。在本研究中，我们分析了UCBT和外周血干细胞移植（PBSCT）两种移植类型淋巴细胞重建与慢性GVHD（cGVHD）发生的相关性。

## 病例与方法

1. 病例：本研究纳入2018年3月至2019年8月在中国科学技术大学附属第一医院血液科接受allo-HSCT的血液病患者124例，入组标准：①免疫重建过程具有连续检测记录；②接受非去T细胞为预处理方案；③中性粒细胞植入。其中非血缘UCBT 96例，PBSCT 28例。男61例，女63例，中位年龄为20（1～64）岁。按照2014年美国国立卫生研究院（NIH）提出的等级标准[8]进行cGVHD的评估和分类。

2. 移植预处理方案：PBSCT组采用的预处理方案包括：①白消安（Bu）+环磷酰胺（Cy）：Bu 3.2 mg·kg^−1^·d^−1^×4 d，Cy 60 mg·kg^−1^·d^−1^×2 d，共2例；②Bu+Cy+阿糖胞苷（Ara-C）：Bu 3.2 mg·kg^−1^·d^−1^×4 d，Cy 60 mg·kg^−1^·d^−1^×2 d，Ara-C 2 g·m^−2^·d^−1^×2 d，共2例；③Bu+Cy+氟达拉滨（Flu）：Bu 3.2 mg·kg^−1^·d^−1^×4 d，Cy 60 mg·kg^−1^·d^−1^×2 d，Flu 30 mg·m^−2^·d^−1^×4 d，共22例；④全身照射（TBI）+Cy+Flu：TBI 4 Gy，Cy 14.5 mg·kg^−1^·d^−1^×2 d，Flu 30 mg·m^−2^·d^−1^×4 d，共2例。

UCBT组预处理方案[9]–[10]包括Bu+Cy+Flu方案93例、TBI+Cy+Flu方案1例、TBI+Cy+Ara-C方案2例。

3. GVHD的预防：两组患者均采用环孢素A（CsA）联合霉酚酸酯（MMF）预防GVHD。从移植前1 d开始CsA 2.5～3.0 mg·kg^−1^·d^−1^，持续静脉注射24 h，维持CsA谷浓度250～300 mmol/L持续20 d，然后改为口服至移植后2个月（CsA谷浓度150～200 mmol/L），依据GVHD、复发和感染等情况综合调整。从移植后1 d开始使用MMF 20～30 mg·kg^−1^·d^−1^，移植后20 d开始减量，未发生GVHD的患者于移植后60 d停用[11]–[12]。

4. 流式细胞术检测淋巴细胞亚群：利用流式细胞术监测移植后1、3、6、9、12个月淋巴细胞亚群的变化，包括CD3^+^、CD4^+^、CD8^+^ T细胞和调节性T细胞（Treg，CD4^+^CD25^+^CD127_low_）、B细胞（CD19^+^）和调节性B细胞（Breg，CD19^+^ CD24_high_ CD38_high_CD27^−^CD5_dim_）。同一份标本分3管分别检测：①TBNK：CD45-PerCP-cy5.5、CD19-APC、CD3-FITC、CD4-PE-Cy7、CD8-APC-Cy7、CD16CD56-PE；②Treg：CD45-PerCP-cy5.5、CD4-FITC、CD25-PE和CD127-APC；③Breg：CD45-PerCP-cy5.5、CD19-PE-Cy7、CD27-FITC、CD24-PE、CD5-APC和CD38-Pacific Blue。用EDTA抗凝管收集外周血2 ml，取200 µl全血分别加入三组荧光素标记的抗体，同时做同型对照及空白对照管，4 °C避光孵育20 min，然后加入红细胞裂解液避光反应5 min，加入PBS洗涤后上机检测，使用Flow Jo v10.0分析流式数据。

5. 统计学处理：使用SPSS 26.0和GraphPad Prism7.0进行统计学处理和绘图，患者临床特征中连续变量使用Mann-Whitney *U*检验，分类变量使用卡方检验或者Fisher精确检验。分析两种移植类型患者在不同时间点淋巴细胞亚群的比例和计数时，不满足正态分布的数据采用Mann-Whitney非参数检验，统计描述采用中位数及*Q*25～*Q*75的四分位间距，*P*<0.05（双侧）为差异具有统计学意义。

## 结果

1. 患者基本资料：124例患者的基本特征如表1所示。其中常见的移植类型是UCBT（77.42％），两种移植类型中急性髓系白血病所占的比例最高，UCBT组为55.2％，PBSCT组为53.6％。以Bu为主预处理方案在两种移植类型中所占的比例分别是96.9％（UCBT）、92.9％（PBSCT）。两种移植类型中，GVHD的预防方案均为环孢素A（CsA）联合霉酚酸酯（MMF）。

**表1:** 124例异基因造血干细胞移植患者的临床资料

临床资料	非血缘脐血干细胞移植（96例）	同胞外周血干细胞移植（28例）	*P*值
性别［例（％）］			0.522
男	49（51.0）	12（42.9）	
女	47（49.0）	16（57.1）	
中位年龄［岁，*M*（范围）］	14（1~64）	29（1~64）	0.004
疾病类型［例（％）］			0.222
急性淋巴细胞白血病	31（32.3）	5（17.9）	
急性髓系白血病	53（55.2）	15（53.6）	
慢性髓性白血病	2（2.1）	1（3.6）	
骨髓增生异常综合征	4（4.2）	6（21.4）	
其他	6（6.3）	1（3.6）	
预处理方案［例（％）］			0.315
Bu为主	93（96.9）	26（92.9）	
TBI为主	3（3.1）	2（7.1）	
有核细胞输注量［×10^7^/kg，*M*（范围）］	3.08（0.52~11.00）	10.18（3.76~49.01）	<0.001
			
CD34^+^细胞输注量［×10^5^/kg，*M*（范围）］	1.78（0.27~8.36）	10.68（2.82~128.7）	<0.001
ABO血型［例（％）］			<0.001
相合	28（29.2）	24（85.7）	
不相合	68（70.8）	4（14.3）	

注：Bu：白消安；TBI：全身照射

2. cGVHD发生情况：UCBT组16例患者发生cGVHD，其中10例为中重度cGVHD；PBSCT组9例患者发生cGVHD，其中8例为中重度cGVHD。UCBT组、PBSCT组移植后1年cGVHD累积发生率差异无统计学意义［13.54％（95％ *CI* 6.42％～20.12％）对28.57％（95％*CI* 9.72％～43.50％），*P*＝0.057］，但UCBT组中重度cGVHD累积发生率低于PBSCT组［9.38％（95％*CI* 3.35％～15.02％）对28.57％（95％ *CI* 9.72％～43.50％），*P*＝0.008］（图1A、B）。UCBT组两年cGVHD累积发生率和中重度cGVHD累积发生率均低于PBSCT组［15.60％（95％*CI* 9.20％～23.60％）对32.10％（95％*CI* 15.80％～49.70％），*P*＝0.047；10.40％（95％ *CI* 5.30％～17.50％）对28.60％（95％ *CI* 13.30％～46.00％），*P*＝0.014］。

**图1:**
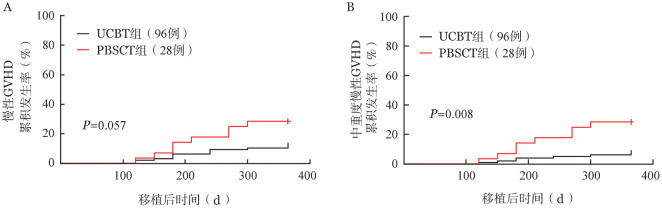
脐血干细胞移植（UCBT）组和同胞外周血干细胞移植（PBSCT）组慢性移植物抗宿主病（GVHD）（A）和中重度慢性GVHD（B）发生曲线

3. UCBT组与PBSCT组T细胞亚群重建：PBSCT组CD3^+^T细胞计数在移植后第1个月高于UCBT组［79.00（38.00～132.70）×10^7^/L对39.70（21.18～83.93）×10^7^/L，*P*＝0.009］，第6个月后UCBT组超过PBSCT 组。UCBT组CD8^+^T细胞和Treg细胞计数与PBSCT组差异无统计学意义。UCBT组CD4^+^T细胞计数在移植第3个月后始终高于PBSCT组，移植后第6、9和12个月差异有统计学意义（表2）。

**表2:** 移植后淋巴细胞亚群计数变化［×10^7^/L，*M*（范围）］

移植后时间（月）	UCBT组（96例）	PBSCT组（28例）	*z*值	*P*值
CD3^+^细胞				
1	39.70（21.18～83.93）	79.00（38.00～132.70）	2.624	0.009
3	98.80（67.60～151.40）	106.05（65.80～170.28）	0.416	0.677
6	165.30（92.85～228.50）	115.00（97.10～168.40）	1.855	0.064
9	174.10（121.85～259.95）	145.70（106.73～209.45）	1.341	0.180
12	188.25（128.95～272.95）	165.95（121.45～220.10）	1.248	0.212
CD4^+^细胞				
1	12.00（7.35～19.95）	13.50（8.70～22.90）	1.347	0.178
3	30.30（19.40～48.00）	23.20（14.32～36.22）	1.909	0.056
6	59.00（36.70～89.65）	31.40（18.10～44.00）	4.279	<0.001
9	71.30（49.60～101.45）	41.60（25.82～56.27）	4.466	<0.001
12	83.00（50.17～121.55）	44.85（31.62～62.10）	4.095	<0.001
CD8^+^细胞				
1	19.15（4.97～50.15）	26.50（12.30～62.80）	1.674	0.094
3	61.3（33.80～100.01）	58.50（36.40～110.40）	0.114	0.910
6	93.10（49.45～130.60）	67.70（39.70～109.40）	1.228	0.219
9	87.20（53.65～162.15）	84.70（67.82～142.50）	0.281	0.779
12	90.20（58.35～144.25）	94.50（66.95～142.87）	0.576	0.565
Treg细胞				
1	0.28（0.08～0.68）	0.55（0.27～0.75）	2.101	0.061
3	1.22（0.47～2.09）	1.17（0.79～1.56）	0.095	0.925
6	1.41（0.89～2.36）	1.86（0.97～2.53）	0.941	0.347
9	2.19（1.38～3.49）	1.92（0.91～3.46）	0.722	0.470
12	3.11（1.76～5.06）	2.54（1.45～4.24）	1.010	0.313
B细胞				
1	0.10（0～0.30）	0.70（0.30～1.70）	4.343	<0.001
3	2.10（0.06～13.10）	5.45（1.80～7.65）	1.016	0.309
6	28.50（6.75～52.95）	11.10（4.10～34.90）	1.826	0.068
9	53.80（28.00～103.20）	23.35（5.07～35.00）	4.319	<0.001
12	66.70（36.97～98.72）	20.85（7.72～39.40）	4.677	<0.001
Breg细胞				
1	0	0	3.322	0.001
3	0.13（0～1.04）	0.02（0～0.24）	1.376	0.169
6	1.23（0.38～3.52）	0.05（0～0.84）	3.599	<0.001
9	2.25（1.07～6.71）	0.12（0～0.77）	5.043	<0.001
12	3.69（0.83～8.66）	0.46（0～0.93）	5.046	<0.001

注：UCBT：脐血干细胞移植；PBSCT：外周血干细胞移植；Treg细胞：调节性T细胞；Breg细胞：调节性B细胞

PBSCT组CD3^+^T细胞比例始终高于UCBT组，移植后第12个月差异具有统计学意义［69.15％（59.95％～78.35％）对60.25％（54.53％～70.93％），*P*＝0.011］。PBSCT组CD8^+^T细胞比例在移植后第9和12个月高于UCBT组［41.99％（34.24％～50.40％）对31.24％（22.28％～41.19％），*P*<0.001；39.79％（34.47％～47.24％）对29.73％（22.40％～39.05％），*P*<0.001］。UCBT组CD4^+^T细胞比例始终高于PBSCT组（*P*<0.05），PBSCT组Treg细胞比例始终高于UCBT组（*P*<0.05）（表3）。

**表3:** 移植后淋巴细胞亚群比例变化［％，*M*（范围）］

移植后时间（月）	UCBT组（96例）	PBSCT组（28例）	*z*值	*P*值
CD3^+^细胞				
1	61.25（47.00～75.85）	63.65（54.28～73.83）	0.481	0.630
3	69.95（59.33～77.18）	73.90（61.78～77.88）	0.873	0.383
6	68.40（58.35～77.73）	70.30（58.98～75.50）	0.024	0.981
9	62.80（50.68～72.73）	68.00（58.35～75.30）	1.596	0.111
12	60.25（54.53～70.93）	69.15（59.95～78.35）	2.528	0.011
CD4^+^细胞				
1	18.00（10.40～27.98）	13.95（9.60～18.08）	2.352	0.019
3	21.15（15.30～27.05）	14.25（11.15～18.43）	3.583	<0.001
6	26.60（19.55～32.18）	18.10（12.10～22.00）	4.500	<0.001
9	25.65（21.68～29.53）	18.70（13.95～21.48）	4.844	<0.001
12	26.25（21.13～30.98）	19.55（14.68～24.68）	3.583	<0.001
CD8^+^细胞				
1	30.85（15.17～49.34）	28.14（17.11～46.17）	0.131	0.895
3	39.63（32.19～50.40）	38.78（30.75～47.00）	0.341	0.733
6	38.27（26.27～45.08）	38.09（32.18～48.80）	0.777	0.437
9	31.24（22.28～41.19）	41.99（34.24～50.40）	3.786	<0.001
12	29.73（22.40～39.05）	39.79（34.47～47.24）	3.825	<0.001
Treg细胞				
1	2.35（1.20～4.48）	4.10（2.40～5.38）	2.502	0.012
3	3.60（2.03～5.50）	4.70（3.13～6.58）	2.011	0.044
6	2.80（1.63～4.75）	5.95（4.93～7.98）	5.326	<0.001
9	3.60（2.23～4.90）	5.35（4.20～7.16）	3.865	<0.001
12	4.10（3.13～5.60）	5.25（4.43～7.65）	3.447	0.001
B细胞				
1	0.20（0.10～0.40）	0.45（0.30～2.20）	3.162	0.002
3	1.40（0.45～8.15）	3.45（1.55～4.20）	1.345	0.178
6	12.75（3.38～22.60）	7.05（2.95～15.03）	1.877	0.061
9	21.45（11.80～30.45）	9.00（3.08～16.73）	4.449	<0.001
12	22.20（14.93～29.68）	8.75（5.80～18.93）	5.020	<0.001
Breg细胞				
1	0	0	3.242	0.001
3	1.00（0～12.55）	0.10（0～4.83）	1.460	0.144
6	5.35（1.90～12.20）	1.45（0～7.78）	3.051	0.002
9	6.25（2.00～12.33）	0.80（0～5.25）	3.608	<0.001
12	6.15（1.63～11.75）	1.40（0.18～5.85）	3.310	<0.001

注：UCBT：脐血干细胞移植；PBSCT：外周血干细胞移植；Treg细胞：调节性T细胞；Breg细胞：调节性B细胞

4. B细胞亚群的重建：造血干细胞移植后，B细胞在计数和比例上的重建趋于一致。PBSCT组B细胞计数和比例在移植后的第1个月均高于UCBT组；第6个月之后，UCBT组计数和比例高于PBSCT组，移植后第9和12个月具有统计学差异，详见表2、表3。

造血干细胞移植后，Breg细胞在计数和比例上的重建趋势一致。UCBT组Breg细胞计数和比例始终高于PBSCT组，移植后第6、9、12个月差异具有统计学意义，详见表2、表3。

5. UCBT与PBSCT组B细胞亚群与cGVHD的关系：UCBT后，非cGVHD组的B细胞计数和比例始终高于中重度cGVHD组，两组的B细胞计数在移植后第6和12个月差异具有统计学意义［29.05（6.95～54.90）×10^7^/L对7.00（1.05～14.45）×10^7^/L，*P*＝0.038；70.40（40.10～99.04）×10^7^/L对26.20（17.15～68.50）×10^7^/L，*P*＝0.043］；两组B细胞比例在移植后第6个月也差异具有统计学意义［14.00（3.75～24.67）％对3.70（0.85～8.62）％，*P*＝0.049］。（图2A、2B）。UCBT后，非cGVHD组的Breg细胞计数和比例始终高于轻度cGVHD组和中重度cGVHD组。非cGVHD组的Breg细胞计数在移植后第6、9、12个月高于中重度cGVHD组［1.58（0.41～4.75）×10^7^/L对0.05（0～0.71）×10^7^/L，*P*＝0.006；2.52（1.25～7.03）×10^7^/L对0.34（0.01～2.12）×10^7^/L，*P*＝0.028；4.11（1.01～9.03）×10^7^/L对0.82（0.33～2.99）×10^7^/L，*P*＝0.050］（图2C）；非cGVHD组的Breg细胞比例在移植后第9个月与中重度cGVHD组差异具有统计学意义［6.65％（2.45％～11.77％）对1.20％（0.23％～9.00％），*P*＝0.038］（图2D）。PBSCT组非cGVHD患者B细胞计数和比例与中重度cGVHD患者相比差异无统计学意义。PBSCT组非cGVHD患者的Breg细胞计数和比例与中重度cGVHD患者比较差异无统计学意义。

**图2:**
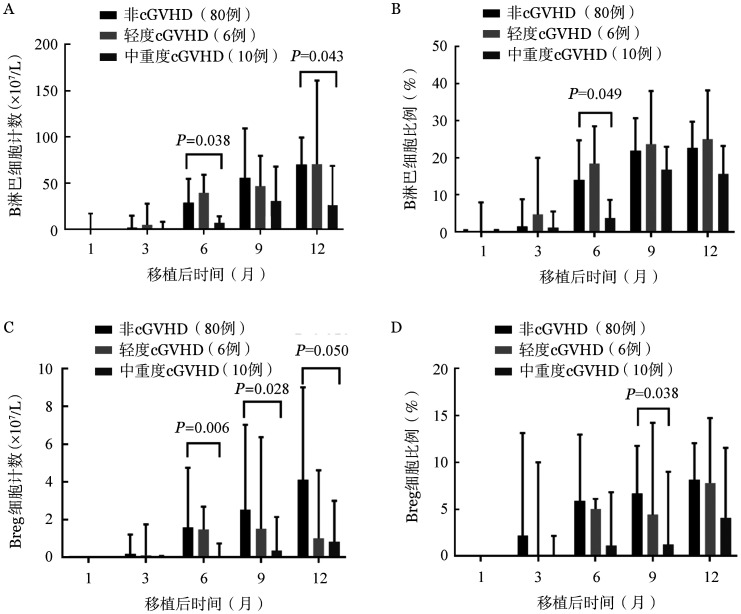
非血缘脐血干细胞移植后非慢性移植物抗宿主病（cGVHD）组、轻度cGVHD组和中重度cGVHD组的B细胞亚群重建情况 A：B淋巴细胞计数；B：B淋巴细胞比例；C：调节性B细胞（Breg）计数；D：Breg细胞比例

## 讨论

恶性血液病患者可以通过allo-HSCT获得长期的存活，但长期存活的部分患者可发生cGVHD，是非复发死亡的主要原因[13]–[14]。cGVHD的特征是纤维化，具有硬皮病样变化，类似于自身免疫性疾病[15]。影响cGVHD的因素包括HLA的差异、供受者的年龄、病毒感染、预处理方案以及免疫细胞的重建等[16]。移植后的免疫重建分为多个阶段，固有免疫细胞先恢复，然后是适应性免疫细胞的恢复[17]–[18]。固有免疫细胞包括单核细胞、粒细胞、树突状细胞（DC）和自然杀伤细胞（NK），通常在移植后的最初几周至几个月内恢复。适应性免疫细胞包括T淋巴细胞和B淋巴细胞，重建过程较慢，一般需要1年到2年的时间。

allo-HSCT后T细胞重建有两种模式，一种是在移植后的早期，供者来源的T细胞与受者体内残留的T细胞在受者外周免疫器官中扩增；另一种发生过程相对较晚，是由供者来源的淋巴祖细胞或者造血干细胞在胸腺中发育而成[19]–[20]。在本研究中，我们用流式细胞术首先分析了UCBT和PBSCT两种移植类型的患者淋巴细胞重建情况，发现两种移植类型在T细胞亚群重建上的差异。在CD4^+^T细胞免疫重建过程中，UCBT组的计数和比例高于PBSCT组，这与Jacobson等[21]的研究结果不一致，产生差异的主要原因可能是本中心采用了不含抗胸腺细胞球蛋白（ATG）的预处理方案，有效保护了脐血中的T细胞，从而移植后有大量不依赖胸腺的T细胞在外周扩增。两种移植方式在B细胞亚群重建上也存在差异。在B细胞免疫重建的初期，由于PBSCT组的有核细胞输注量要高于UCBT组，导致PBSCT组的计数和比例均高于UCBT组；在免疫重建的后期，UCBT组远高于PBSCT组。在Breg细胞免疫重建过程中，UCBT组的计数和比例均高于PBSCT组。以往的研究证实，UCBT在B细胞恢复方面优于PBSCT是因为脐血含有较高比例的祖细胞而具有更好的体外和体内B淋巴细胞重建能力[22]–[23]。这些结果提示，UCBT和PBSCT之间免疫细胞重建过程有一定差异，这种差异可能是两种移植类型的cGVHD发生率不同的原因。

造血干细胞移植后，非cGVHD组B细胞和Breg细胞的计数和比例在移植后的前三个月较低，随后逐渐回升。本研究中，UCBT和PBSCT患者中非cGVHD组Breg细胞计数和比例均高于中重度cGVHD组，表明Breg细胞可以显著降低cGVHD的发生。Khoder等[24]在研究中同样发现了allo-HSCT后，cGVHD患者体内Breg细胞比例较非cGVHD患者和正常人降低，并且产生调节因子IL-10较少。而本中心前期研究显示，脐血中Breg细胞计数和比例显著高于外周血，这可能是UCBT cGVHD发生率低于PBSCT的原因[25]–[27]。

本研究结果显示，UCBT和PBSCT患者淋巴细胞重建存在差异，这种差异可能与两种移植类型中cGVHD的发生有关。该数据分析将有助于了解抑制cGVHD的发病机制，并为开发更有效的cGVHD治疗策略提供线索。因此，接下来的基础研究中，需要找到相关分子靶点，探究UCBT后Breg细胞重建较快的原因及其对cGVHD的调控机制。
